# Use of complementary therapies and supportive measures of patients with intracranial gliomas—a prospective evaluation in an outpatient clinic

**DOI:** 10.1007/s11060-024-04696-1

**Published:** 2024-05-06

**Authors:** Malte Ottenhausen, Mirjam Renovanz, Isabell Bartz, Alicia Poplawski, Darius Kalasauskas, Harald Krenzlin, Naureen Keric, Florian Ringel

**Affiliations:** 1 Department of Neurosurgery, University Medical Center Mainz, Mainz, Germany; 2 Department of Neurology & Interdisciplinary Neuro-Oncology, Hertie Institute for Clinical Brain Research, Eberhard-Karls-University of Tübingen, Tübingen, Germany; 3 Center for Neuro-Oncology, Comprehensive Cancer Center Tübingen-Stuttgart, University Hospital Tübingen, Eberhard Karls University, Tübingen, Germany; 4 Department of Neurosurgery, University Hospital Tübingen, Tübingen, Germany; 5 Institute of Medical Biostatistics, Epidemiology and Informatics (IMBEI), University Medical Center of the Johannes Gutenberg University Mainz, 55131 Mainz, Germany

**Keywords:** Glioma, Supportive care, Complementary therapies, CAM

## Abstract

**Purpose:**

Patients with intracranial gliomas frequently seek for complementary and alternative medicine (CAM), in addition to guideline-directed therapy. In this study, we therefore assessed patients’ information needs regarding treatment and support, and evaluated their attitudes toward experimental trials and alternative therapies.

**Methods:**

A prospective, cross-sectional, descriptive survey was conducted in our center. We developed an interview focusing on how patients obtain further information about therapy and the use of alternative/complementary therapies.

**Results:**

A total of 102 patients participated in the survey. 50% (*n* = 51) of patients reported that they had not attempted any additional therapies. When patients attempted self-therapy, it was most commonly in the areas of nutrition (25%, *n* = 26) and dietary supplements (17%, *n* = 17). Alternative or complementary therapies were used by 14% (*n* = 14) of the patients. Younger age (Odds ratio (OR) 0.96 (95% Confidence interval (CI) 0.92–0.99, *p* = 0.012) and tumor entity (OR 5.01 (95% CI 1.66–15.11, *p* = 0.004) for grade 4 vs. 3 tumors and OR 7.22 (95% CI 1.99–26.28) for grade 4 vs. other tumors *p* = 0.003) were significantly associated with a greater interest in CAM.

**Conclusions:**

Interest in complementary and alternative medicine, as well as nutrition and dietary supplements is high (51%) among glioma patients, and significantly higher among younger patients and those with a worse diagnosis (WHO grade 4). A comprehensive approach to information, including paramedical topics, is needed to provide optimal patient counseling and care for glioma patients.

**Supplementary Information:**

The online version contains supplementary material available at 10.1007/s11060-024-04696-1.

## Introduction

Gliomas are the most common primary intracranial tumors, accounting for more than 80% of malignant brain tumors. These tumors are highly diverse, and their prognosis varies widely: overall survival for patients with pilocytic astrocytoma can be over 90% at five years, while it is less than 5% for glioblastoma [1]. High-grade gliomas (WHO grade 3 and 4) account for 75% of all gliomas [2].

First-line therapy for most cases of high-grade gliomas is maximum safe resection, followed by concomitant radiochemotherapy and adjuvant chemotherapy with temozolomide [3]. Treatment for progression is much less standardized and ranges from re-resection and/or radiation- and chemotherapy to supportive care [4]. In addition to the standard of care, recent advances such as biomarker-based therapy [5], immunotherapy [6], and recombinant viruses [7] are currently only being evaluated in clinical trials.

After being diagnosed with glioma, patients are faced with an overwhelming amount of information. Educating patients and their families about the disease and treatment options, as well as identifying those patients who need supportive care, is an important part of glioma care and at the same time challenging [8].

Neurological deficits, seizures, and treatment side effects are often accompanied by psychological distress [9, 10], fatigue [11] and depression [12]. In addition, patients and caregivers may suffer from a reduced quality of life, financial difficulties, burnout symptoms, and feelings of inadequate information [13]. Early palliative care interventions can improve symptom control and quality of life in these patients [14]. The importance of supportive care increases as the disease progresses and patients lose cognitive and decision-making abilities.

During the course of the disease many patients seek for complementary and alternative medicine (CAM), such as dietary modification [15], herbal medicine [16], or opioids [17], in addition to standard clinical therapy in the hope of improving their prognosis and combating treatment side effects and neurological deficits caused by the disease. It is worth noting that the definition of CAM varies across the literature. For our study, we followed the definition provided by the National Center for Complementary and Integrative Health (NCCIH) and included all therapies that were used in addition to standard therapy [18].

Lack of information and unmet needs concerning CAM can lead to patient and caregiver dissatisfaction and inappropriate use of alternative approaches [9]. Therefore clinicians should be prepared and willing to help patients navigate different treatment options. The question remains as how to assess whether enough information is being provided. Specific tools to assess supportive care needs are needed for glioma patients, as cognitive deficits pose significant challenges [8].

In this study, we assessed patients’ information needs regarding treatment and support, and evaluated patients’ attitudes toward experimental trials and alternative therapies.

## Methods

A prospective, cross-sectional, descriptive survey was conducted at our center in 2020. We developed an interview based on literature research and a post-hoc analysis of a multicenter trial [19, 20], including 10 questions focusing on how patients obtain further information about therapy, support, and the use of alternative/complementary.

### Inclusion criteria

- Diagnosis of a high-grade glioma or other brain tumor.
- Sufficient language comprehension was required for inclusion in this study.
- Patients over 18 years of age were contacted during their postoperative therapy *or follow-up.*

Patients with extremely impaired general condition and neurocognitive impairment who were unable to complete the questionnaire even with the help of a personal assistant were excluded. The Research Ethics Committee of Rhineland-Palatinate has confirmed that no ethical approval is required (No *2020–14,935*). Informed consent was obtained from all individual participants included in the study.

### Interview procedure

Patients were interviewed in the outpatient clinic with the assistance of study staff, who had received detailed training. The interviewer was not involved in the clinical care of the participants and was therefore introduced to the participants. Interviews were conducted in a separate room and in the absence of any other participants. Study subjects were interviewed face-to-face and in German language. The interviews were then analyzed by MO and IB. The interview questions for the patients are shown in the supplements.

### Data analysis

The notes from the interviews were sorted into domains and main and subordinate contents. Qualitative data is reported in accordance with the Consolidated Criteria for Reporting Qualitative Research (COREQ) guidelines. The results were summarized using frequency counts, data were analyzed descriptively.

The pseudonymized data were imported into Excel and SPSS (Statistical Package for the Social Sciences). Coding by dichotomous variables was chosen for all multiple responses. Analysis was performed using Excel and SPSS version 23. Descriptive analysis was performed for each question separately. Logistic regression was used to assess the influence of different variables on interest in complementary therapies. The diagnosis group was divided into three groups according to prognosis. Group 1 included all CNS WHO grade 4 tumors, group 2 included all CNS WHO grade 3 tumors, and group 3 included various benign tumors. Age was included in the analyses as a continuous variable. The significance level was set at 5%.

## Results

### Patients characteristics

A total of 107 patients were contacted and 102 participated in the survey, 2 patients refused and 3 patients were excluded from the survey. One patient was excluded due to a lack of German language skills, one patient was treated in another clinic and only came to our clinic for a second opinion, and one patient was diagnosed for the first time. The participation rate was 98.08%. The mean age of the respondents was 53+/-15 years. Of the respondents, 41% (*n* = 42) were female and 59% (*n* = 60) were male.

Glioblastoma (CNS WHO grade 4) was the most common diagnosis, accounting for 38% (*n* = 19)1(Table 1).

**Table 1:** Frequencies of different diagnoses

Diagnosis	*N*	%
Glioblastoma	39	38
Anaplastic Astrocytoma	23	23
Anaplastic Oligoastrocytoma	8	8
Anaplastic Oligodendroglioma	6	6
Other	26	25

When asked about molecular pathologic features of the tumor, 82.4% of patients (*n* = 84) did not know any of the listed markers.

### Information needs

16% of participants (*n* = 16) did not know their own diagnosis. Source of information about the tumor disease was the treating neurooncologist/surgeon for 91% (*n* = 93), the Internet for 58% (*n* = 59), and the general practitioner for 53% (*n* = 54) of respondents. A naturopath/alternative practitioner was consulted by 7% (*n* = 7).

10% (*n* = 10) of respondents selected the category “other”. Other sources of information included magazines/brochures (*n* = 3), books (*n* = 2), visit to a bio-oncologist (*n* = 2), visit to a patient education symposium on the World Brain Tumor Day (*n* = 1), a self-trained alternative practitioner (*n* = 1), homeopaths (*n* = 2), and a radiotherapist (*n* = 1). Two patients indicated that they had no need for information and did not use any of the sources mentioned. 50% (*n* = 51) of patients reported that they had not attempted any additional therapies. Patients self-therapy attempts are shown in Table 2. Seven patients (7%) selected “other” to describe their own therapy attempts, including selenium supplementation and regular bowel cleansing.

Patient’s information needs are shown in Table 3. Overall, 32% of patients (*n* = 33) reported feeling adequately informed by the services provided during the consultation

**Table 2:** Frequencies of self-therapy attempts

Self-therapy attempt	Responses	
	*N*	%
Nutrition (eat healthier or eat special foods e.g. nuts)	26	25
Special diet (e.g. ketogenic diet)	3	3
Sport program	17	17
Nutritional supplements	17	17
Alternative therapies	14	14
Other	7	7
None	51	50

**Table 3:** Frequencies of the need for information

Need for Information	Responses	
	*N*	%
Current studies	39	38
Support groups	5	5
Sports	25	24
CAM	44	43
Psychological support	28	27
Other	7	7
None	33	32

63% (*n* = 64) reported that they had not sought a second opinion at the time of the survey. 34% (*n* = 35) of the patients who did seek a second opinion most frequently reported that they contacted a second neurosurgeon or neurosurgical department. The neurologist and the oncologist were the second most common contacts with 3% each (*n* = 3). Only one patient sought a second opinion from an alternative physician. When asked whether and from whom they had received information about current medical trials, 44% (*n* = 45) of patients said that they had been informed about current medical trials by their physician. The Internet was used as a source of information by 11% (*n* = 11) of respondents. 46% (*n* = 47) reported that they did not receive information about current trials. Overall, 72% (*n* = 73) of respondents could imagine participating in a medical trial, and 21% (*n* = 21) could even imagine taking a higher risk.

### Logistic regression analysis

**Fig. 1:**
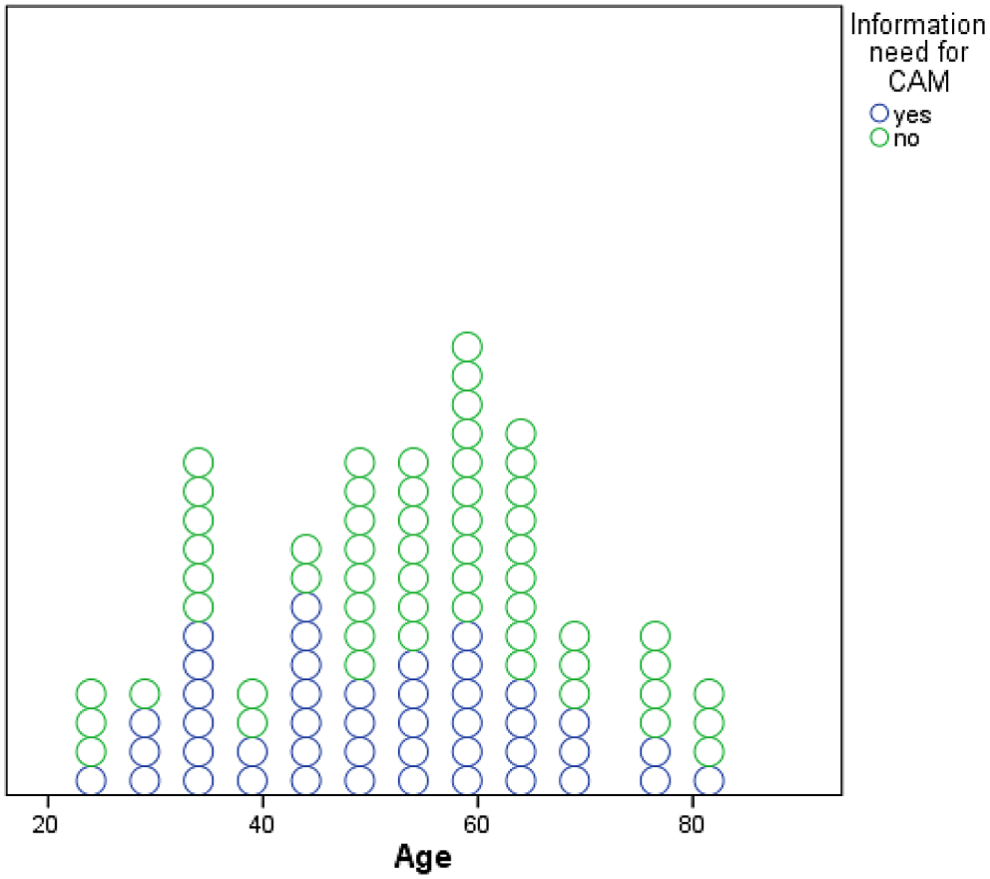
Relationship between information needs according to CAM and age

To evaluate the factors associated with the need of information about CAM, we performed a multivariate logistic regression analysis, and included patient age, gender and tumor entity as independent variables. Younger age (Odds ratio (OR) 0.96 (95% Confidence interval (CI) 0.92–0.99, *p* = 0.012) and tumor entity (OR 5.01 (95% CI 1.66–15.11, *p* = 0.004) for grade 4 vs. 3 tumors and OR 7.22 (95% CI 1.99–26.28) for grade 4 vs. other tumors *p* = 0.003) were significantly associated with unmet information needs about CAM (Fig. 1).

Accordingly, patients with grade 4 Tumors have a higher interest in CAM than patients with grade 3 and other tumors. There was no difference between grade 3 and other tumors (Table 4.). Gender (female vs. male) was marginally insignificant with more females expressing unmet information need for CAM (OR 2.37 (95% CI 0.98–5.75, *p* = 0.06).

**Table 4:** Presentation of significant variables with p-value and odds ratio

	*p*-Value	OR	95% CI
**Gender (female vs. male)**	0.06	2.37	0.98–5.75
Age (year)*	0.01	0.96	0.92–0.99
WHO Grade 4 vs. 3*	0.003	7.22	1.99–26.28
WHO Grade 4 vs. other*	0.004	5.01	1.66–15.11
**WHO Grade 3 vs. other**	0.54	1.44	0.45–4.61

## Discussion

We conducted a prospective, cross-sectional, descriptive survey of 102 patients using a 10-question interview to learn more about patients’ attitudes toward information needs and alternative and complementary therapies.

In the survey, 41% of patients reported using complementary and alternative medicine (CAM) in addition to standard clinical therapy, which is consistent with findings from other studies reporting rates between 29% and 77% [21–31].

The utilization of CAM associated to geographic location and diagnosis. Studies have shown that individuals with Chinese heritage (55%) [26] and individuals from the United States (77%) [32] exhibit a greater use of complementary and alternative medicine (CAM) as compared to those treated in Europe (40%) [21]. In a study of glioma patients conducted in Germany, 40% of patients reported using CAM [21]. This figure is significantly lower than the results from the USA, where 77% of patients with glioblastoma surveyed reported using CAM [25].

In our study we found statistically significant relations between age, diagnosis, and the desire to use CAM. According to our results, younger patients and patients with a worse diagnosis (WHO Grade 4) were significantly more likely to use CAM. Gender (female vs. male) was *marginally insignificant* with more females expressing unmet information need for CAM.

Patients with a WHO grade 4 tumor cannot be offered curative therapy, which may explain the increased need to try complementary therapy methods in this group. Due to the poor prognosis, patients might also be more willing to accept risks. Another reason for use may be the desire to alleviate side effects of therapy. A relationship between diagnosis and the use of CAM was also found in other studies [22, 24].

A possible reason why younger patients are more likely to use CAM may be their procurement of information. In our survey, 74% of ≤ 45-year-olds used the Internet as a source of information, compared to 51% of > 45-year-olds.

The data on information retrieval are consistent with the findings of Rudolph et al. and Heese et al. [21, 32]. A relationship between age and CAM use was also found in other studies [21, 24–26, 30].

Why women have a higher interest in CAM may be explained by better communication and information gathering. In addition, it could be postulated that women have different self-care behaviors and use health services more frequently [33]. Thus, in our study, of all the sources of information listed in the survey, 60% of women and 40% of men used more than two sources of information about their illness. This conclusion was also reached by Firkins et al. and Heese et al. [21, 30] A relationship between gender and users was also found in the USA in a survey of 470 GB patients [25].

In our survey, 51% of the patients interviewed wished to be better educated about CAM and nutrition/nutritional supplements in the neuro-oncology consultation, which is higher than the number of actual users (41%). This trend was also observed in other studies [34, 35] highlighting a great need for information about CAM in the broad patient group. Armstrong et al. found that of CAM users surveyed, a full 74% did not discuss use with their physician [31]. Reasons for this action behind the back of the practitioner may be multifaceted.

However, patients named the neurosurgeon as the most frequent contact for general information about the disease (91%), this high percentage can be explained by the fact that the survey was conducted in the neurosurgical consultation. Although 41% of patients used CAM, only 7% of respondents named an alternative practitioner, supporting the theory that much information about CAM comes from friends, family, or the Internet.

The fact that, despite easy access to a variety of media, most patients still cite the face-to-face conversation with the specialist as the first and most important source of information suggests that many of the patients place great trust in medical treatment and feel in good hands. Specialists could use this to recommend suitable sources of information on the Internet if required by the patient.

In our survey, only 32% of patients said they felt sufficiently informed about their disease. Thus, there is a high need for additional information among the respondents. In a 2010 study, face-to-face interviews with patients with HGG revealed that there was a particular need for information regarding diagnosis and prognosis [36]. Due to the severe course of the disease and the accompanying physical and cognitive impairment, it can be assumed that the need for information of patients with glioma is particularly high.

There is a high need for information in the areas of CAM (43%), current studies (38%), exercise (25%), nutrition (28%), and psychological support (28%).

According to Halkett et al. there are large differences in the information needs of glioma patients [36]. Some would like to know exactly how their situation is, other patients would prefer not to know anything at all unless it is positive information.

Accordingly, their level of information also varies. It is worth mentioning that in our survey 15,7% did not know their own diagnosis, which supports this theory. Furthermore, 82% had no molecular pathology knowledge about their tumor. These variations in information needs pose a challenge for the practitioner; additionally, cognitive limitations often limit communication with HGG patients. Nevertheless, it is true that individualized information is important for patient satisfaction and the doctor-patient relationship.

Barriers remain in recruiting patients to clinical trials. These include, for example, failure to approach appropriate patients or their negative attitudes and information gaps. Some patients may think that participation puts them at high risk for successful therapy. For example, in a survey in Oman, less than 1/3 of respondents knew what clinical trials even were, and why they were designed [37].

In Germany and other Western countries, however, there are significantly more studies available than in Oman, which also suggests a higher awareness among patients. In our survey, 72% of respondents could imagine participating in current clinical trials, 21% even at higher risk. This result is in line with that of other studies, although the aforementioned studies did not specifically survey brain tumor patients, but oncology patients in general [37–40].

In our study, 44% of the respondents had been informed by their treating physician about the possibility of participating in current clinical trials, but almost half stated that they had not received any information since the onset of their disease which may be due to the fact that participation in a study is mainly considered after progression after standard therapy.

Nevertheless it may be helpful to address this topic earlier to educate patients about the process and remove possible prejudices.

In our survey, 63% of respondents did not obtain a second opinion. However, those who did obtain a second opinion most often did so at another neurosurgical center. In the literature, this value varies between 7 − 36% [41, 42], but there is no data specifically analyzing glioma patients available. The reason why our result of 37% is relatively high compared to the literature may be due to the fact that a second opinion is explicitly recommended by the treating physicians as obtaining a second opinion can help patients gaining reassurance about their treatment.The results of our study show that many brain tumor patients would like to have more information about CAM. Providing information to patients and at the same time generating more high-quality data (studies) on CAM should become a greater focus for tumor centers. Appropriate professorships or specially trained staff could work on this topic across disciplines in the future.

### Limitations

Several issues limit the quality and generalizability of the data collected, for example, the questionnaire used is not a validated instrument, and the time between surgery and the survey was not recorded. Some questions about CAM also remain unanswered, such as whether patients who use CAM have experienced an improvement in their condition as a result, and the extent to which financial aspects play a role.

## Conclusion

Interest in complementary and alternative medicine as well as nutrition and dietary supplements is high (51%) among patients with glioma. Of the patients surveyed, 41% reported using CAM in addition to standard therapy. In this study, we could show that interest in complementary therapies is significantly higher among younger patients. Interest in complementary medicine is also significantly higher when the diagnosis is worse (WHO grade 4). A comprehensive approach to information, including paramedical topics, is needed to provide optimal patient counseling and care for glioma patients.

### Electronic supplementary material

Below is the link to the electronic supplementary material.

Supplementary Material 1

## Data Availability

No datasets were generated or analysed during the current study.
