# Supplementary material for: Use of complementary therapies and supportive measures of patients with intracranial gliomas—a prospective evaluation in an outpatient clinic
Source: J Neurooncol. 2024 May 6;168(3):507–13. doi: 10.1007/s11060-024-04696-1 (PMC11186898; doi:10.1007/s11060-024-04696-1)
Supplement: Supplementary file 1 — Supplementary Material 1 [file 11060_2024_4696_MOESM1_ESM.pdf]

No.	Question	Answer options
1.	Are you male or female	Male, female
2.	How old are you?	
3.	What is your Diagnosis?	Glioblastoma, Anaplastic Astrocytoma, Anaplastic Oligo, other
4.	Do you know any other molecular pathological details of your disease?	MGMT, 1p19q, IDH, ATRX, none
5.	Where do you get information about the disease and treatment options?	Neurosurgeon, Oncologist, Neurologist, Internet, Support-Group, alternative Practitioner, general Practitioner, other
6.	What do you use against tumor growth independently of the recommendations of your neurosurgeon?	Nutrition, diet, sports program, food supplements, additional medication, alternative therapies, none, others
7.	Which of the following topics would you like to learn more about during your treatment?	Current trials, Support-Groups, sports, nutrition/diet, alternative therapies, psychological support, other, I am sufficiently informed
8.	Did you get a second opinion during your treatment?	Yes Neurosurgeon, Yes Oncologist, Yes Neurologist, Yes alternative Practitioner, No
9.	Were you informed about current studies and the possibility of participating in them?	Yes - by the doctors, Yes – by my own research, No
10.	Could you imagine participating in a study?	Yes – if the risk stays the same, Yes - even if the risk increases, No

Questions asked in the survey
